# Evaluation of the Cytotoxicity, Genotoxicity and Acute Oral Toxicity of *Thymus longicaulis* subsp. *chaubardii* (Rchb.f.) Jalas

**DOI:** 10.3390/ph18071037

**Published:** 2025-07-12

**Authors:** Ayfer Beceren, Ayse Nur Hazar-Yavuz, Ozlem Bingol Ozakpinar, Duygu Taskin, Ismail Senkardes, Turgut Taskin, Ozlem Tugçe Cilingir-Kaya, Ahmad Kado, Elif Caliskan Salihi, Hatice Kubra Elcioglu

**Affiliations:** 1 Department of Pharmaceutical Toxicology, Faculty of Pharmacy, Marmara University, Istanbul 34854, Türkiye; 2 Department of Pharmacology, Faculty of Pharmacy, Marmara University, Istanbul 34854, Türkiye; ayse.hazar@marmara.edu.tr (A.N.H.-Y.); kubra.elcioglu@marmara.edu.tr (H.K.E.); 3 Department of Biochemistry, Faculty of Pharmacy, Marmara University, Istanbul 34854, Türkiye; ozlem.bingol@marmara.edu.tr; 4 Marmara Pharmacy Drug and Innovative Product Development Unit, Faculty of Pharmacy, Marmara University, Istanbul 34854, Türkiye; duygu.taskin@sbu.edu.tr (D.T.); turguttaskin@marmara.edu.tr (T.T.); elif.caliskan@marmara.edu.tr (E.C.S.); 5 Department of Analytical Chemistry, Faculty of Pharmacy, University of Health Sciences, Istanbul 34668, Türkiye; 6 Department of Pharmaceutical Botany, Faculty of Pharmacy, Marmara University, Istanbul 34854, Türkiye; isenkardes@marmara.edu.tr; 7 Department of Pharmacognosy, Faculty of Pharmacy, Marmara University, Istanbul 34854, Türkiye; 8 Department of Histology and Embryology, School of Medicine, Marmara University, Istanbul 34854, Türkiye; tugce.cilingir@marmara.edu.tr; 9 ISLAB-2 Core Laboratory, Goztepe Prof. Dr. Suleyman Yalcin City Hospital, Istanbul 34722, Türkiye; ahmetkado94@hotmail.com; 10 Department of Basic Pharmaceutical Sciences, Faculty of Pharmacy, Marmara University, Istanbul 34854, Türkiye

**Keywords:** *Thymus longicaulis* subsp. *chaubardii*, cytotoxicity, genotoxicity, acute toxicity

## Abstract

**Background/Objectives**: *Thymus longicaulis* subsp. *chaubardii* (TL) (Rchb.f.) Jalas is widely used in traditional Turkish medicine for respiratory, digestive and uro-genital disorders. The aim of this study was to determine its phytochemical profile and to evaluate its cytotoxic, genotoxic and acute oral toxicity effects. **Methods**: The phenolic composition of the methanolic extract was determined by HPLC-DAD. Cytotoxicity and genotoxicity were evaluated in NIH3T3 cells using MTT, comet and micronucleus assays. Acute toxicity was evaluated in rats at doses of 300 and 2000 mg/kg body weight according to the OECD Guideline 420. **Results**: Rosmarinic acid (87.37 ± 5.39 µg/mg) was the major phenolic compound. TL extract showed >90% cell viability at 50–200 µg/mL, indicating no cytotoxicity. Comet assay revealed a slight increase in DNA damage at 100–200 µg/mL (*p* < 0.001), though significantly lower than the H_2_O_2_ group (*p* < 0.001). No significant (*p* > 0.05) effect was observed in the micronucleus assay between the treated groups. In rats, TL extract caused no mortality or behavioral changes over 14 days. No significant differences were observed in body or organ weights. Hematologically, platelet count increased (*p* < 0.001) and eosinophils decreased (*p* < 0.01 and *p* < 0.001). Biochemical tests showed lower ALT and AST levels (*p* < 0.01 and *p* < 0.05, respectively) and significantly decreased triglycerides in the high-dose group (*p* < 0.001). Histopathological examination showed no organ damage. **Conclusions**: The results of this study indicate that TL methanol extract is non-toxic up to 2000 mg/kg and exhibits no significant cytotoxic or genotoxic effects. These findings support its safe use and traditional medicinal value.

## 1. Introduction

Toxicological studies are particularly important when a plant is considered for broader pharmacological use or for formulation as a phytotherapeutic product. Acute toxicity studies on plants are essential for evaluating their safety profiles, especially given their widespread use in traditional medicine [1]. These studies help identify potential toxic effects and establish safe dosage ranges, ensuring their therapeutic benefits outweigh the risks. Although traditional medicinal plants attract attention in drug discovery studies, they require toxicity assessments due to their side effects with life-threatening consequences [2]. In this way, the safety of their impact on traditional use is evaluated, and integration into drug discovery studies in modern medicine is ensured [3]. Although plants are widely used in traditional medicine for their therapeutic effects, their impact on certain organs (ex. kidneys, liver and bladder) may negatively influence the benefit-risk balance, particularly in individuals with chronic diseases [4]. The fine line between a remedy and a poison was established centuries ago by Paracelsus’s (1493–1541) principle: ‘The dose makes the poison’.

The genus *Thymus* L., consisting of over 200 species, is an aromatic plant with significant effects, widespread traditional use and the focus of extensive research on the Lamiaceae family [5]. Accumulated data indicate that essential oils and bioactive compounds mediate the broad range of activities exhibited by *Thymus* species. One of these, *Thymus longicaulis* subsp. *chaubardii* (Rchb.f.) Jalas [Syn.: *Thymus longicaulis* var. *subisophyllus* (Borbás) Jalas], is widely found in the flora of Türkiye, in S.W., W., N.W., N.E. and C. Anatolia [6]. In addition to its name “kekik”, like other *Thymus* species [7], the plant is also known by the names “çay otu, çobrisa, kekik çayı, kekik otu, keklik otu, yeşil kekik otu, aş kekik, taş kekiği and yer kekiği” in Türkiye [8]. According to the analysis results obtained from *T. longicaulis* subsp. *chaubardii* (TL) leaves and flowers, it has been shown that it contains 92 volatile compounds, and the highest rates of these compounds are methyl (1-methylethyl)-benzene, caryophyllene, thymol, 1,4-cyclohexadiene and carvacrol [9]. The aerial parts and leaves of TL have been reported to be used widely in Anatolia as a folk medicine to relieve shortness of breath, colds, stomachache, indigestion, loss of appetite, flatulence, nausea, diabetes, cholesterol, hypertension, cardiovascular diseases, hemorrhoids, eczema, kidney stones, rheumatic pain, insomnia, anxiety, menstrual pain, toothache and mouth sores, especially in the form of a decoction and infusion [10,11].

Antioxidant activities of hexane, ethyl acetate and methanol extracts prepared from the plant by Sarıkurkcu et al. were investigated by DPPH and reducing power methods. It was found that methanol extract studied at a 0.2 mg/mL concentration had stronger reducing power (absorbance: 0.615 ± 0.010) and DPPH radical scavenging (69.45 ± 0.69%) activities compared to other extracts. It was also determined that methanol extract contained the highest amount of flavonoids (30.11 ± 0.04 μg QEs/mg extract). Therefore, this study reported that the methanol extract of TL exhibited strong in vitro antioxidant activity depending on the dose, and suggests that this activity may be related to the rich flavonoid content of the extract [12].

Although various pharmacological effects of the bioactive components of *T. longicaulis* have been demonstrated in vitro, there is a lack of comprehensive in vivo toxicity data to support its safe application in medicinal formulations. Given its extensive use in folk medicine and high content of bioactive constituents, such as flavonoids and thymol, it is crucial to evaluate its toxicological profile. This is especially important as the therapeutic use of traditional herbal remedies continues to rise, often in parallel with conventional treatments. Therefore, in this study, we aimed to assess the in vivo acute toxicity of *T. longicaulis* methanol extract following the Organization for Economic Co-operation and Development (OECD) guidelines (Test No. 420, fixed-dose method). Additionally, cytotoxicity and genotoxicity assessments were conducted using 3-(4,5-Dimethylthiazol-2-yl)-2,5-Diphenyltetrazolium Bromide (MTT), comet and micronucleus (MN) assays in NIH3T3 cells to provide a comprehensive safety profile.

## 2. Results

### 2.1. Phenolic Compounds

**Figure 1:**
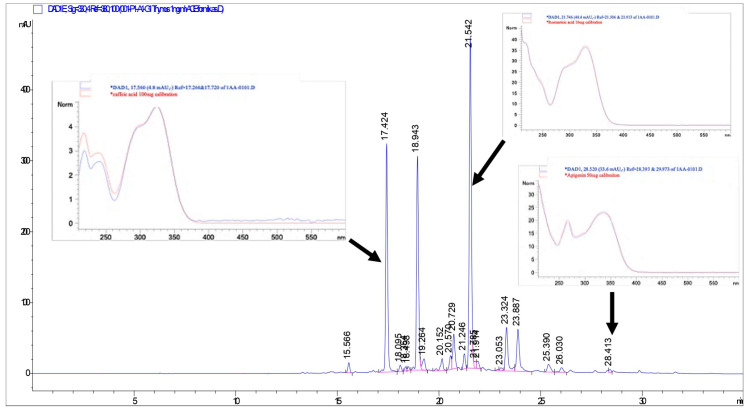
HPLC-DAD chromatogram of TL extract at 330 nm.

The phenolic compounds of the TL extract were identified by high-performance liquid chromatography with diode-array detection (HPLC-DAD), and the results are presented in Table 1. The HPLC-DAD chromatogram of the TL extract and the figures showing the overlap of the spectra of the peaks of the compounds found in the sample chromatogram with the spectra of the standard substances are presented below (Figure 1).

When the high-performance liquid chromatography (HPLC) results are examined from Table 1, it can be seen that the major substance in the TL extract is rosmarinic acid at a level of 87.37 µg/mg.

### 2.2. Cytotoxicity

**Figure 2:**
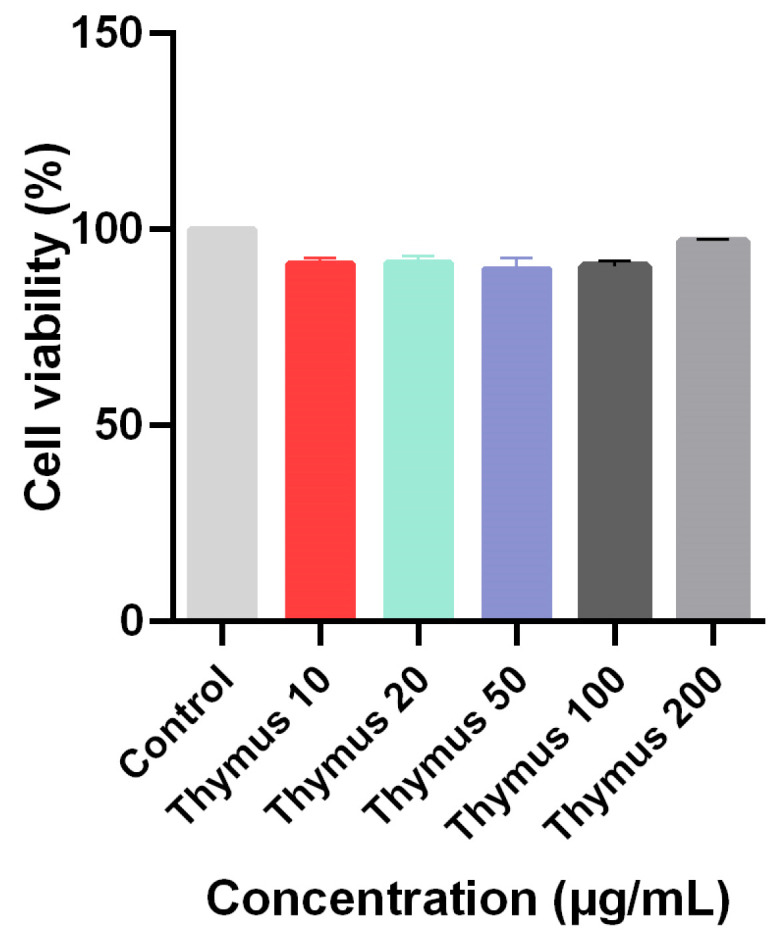
Effect of TL extracts on NIH3T3 cell viability.

The NIH3T3 cell viability percentages after 24 h of treatment of TL extracts were measured. According to the results obtained, it was determined that the cells incubated with TL extracts at the specified concentrations showed over 90% viability. The plant extracts used in this study did not show cytotoxic effects on the NIH3T3 cell line in the applied dose range (Figure 2).

### 2.3. Genotoxicity

#### 2.3.1. Comet Assay Findings

**Figure 3:**
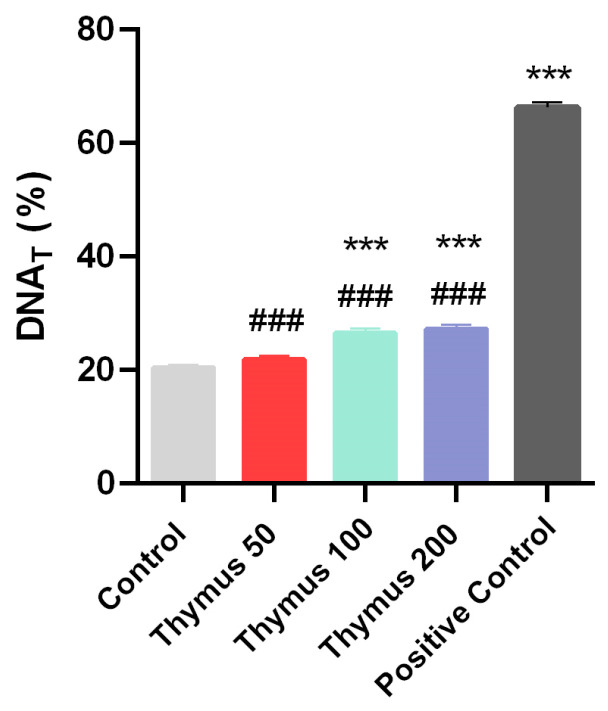
DNA_T_ (%) values assessed using the comet technique. *** *p* < 0.001 compared to control group; ^###^ *p* < 0.001 compared to positive control group. DNA_T_ (%): Percentage of DNA in tail.

As shown in Table 2 and Figure 3, a statistically significant difference (*p* < 0.001) was found after a 24 h application of TL extracts at 100 and 200 µg/mL doses compared to the control, while no statistically significant difference (*p* > 0.05) was found at the lowest dose of 50 µg/mL compared to the control in DNA damage. No significant difference (*p* > 0.05) was found between the 100 and 200 µg/mL doses of TL extracts, and it was observed that the damage did not increase with the increase in dose. Treatment with H_2_O_2_ caused significant DNA damage in NIH3T3 cells. On the other hand, all doses were observed to induce significantly (*p* < 0.001) lower DNA damage in comparison to the positive control cells.

#### 2.3.2. Micronucleus Test

**Figure 4:**
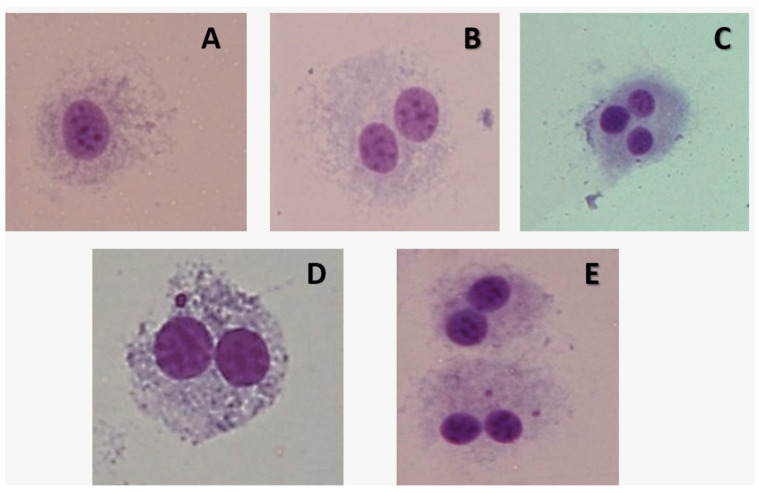
Representative images of the NIH3T3 cells scored in the MN assay. (**A**) Mononucleated cell; (**B**) BN cell; (**C**) multinucleated cell; (**D**) BN cell containing one micronucleus; (**E**) BN cell containing two MNi (Olympus, BX51, Tokyo, Japan, 400×). BN: binucleate cells; MNi: micronuclei; MN: micronucleus.

No significant differences were observed in the frequency of MN in NIH3T3 cells between TL-treated groups after administration (Table 3). However, a significant difference was found at 100 and 200 µg/mL versus the control (** *p* < 0.01 and *** *p* < 0.001, respectively). The positive control group induced a significant increase in the frequency of MN when compared to all treated groups at 24 h (*p* < 0.001) (Figure 4).

According to the CBPI parameter, there is no statistically significant difference (*p* > 0.05) between all concentrations of TL extracts and the control. However, a statistically significant difference (*p* < 0.05) was observed between the control and the positive control value.

### 2.4. Acute Toxicity Study

#### 2.4.1. Sighting Study

No signs of toxicity, such as ataxia (e.g., backward or stomach crawling), tremors, diarrhea, salivation or general weakness were observed at any of the tested dose levels (5, 50, 300 and 2000 mg/kg BW). All animals survived throughout the observation period. Based on these results, dose levels of 300 mg/kg and 2000 mg/kg body weight were selected for use in the main study.

#### 2.4.2. Main Study

Oral administration of TL extracts at fixed doses of 300 and 2000 mg/kg body weight did not induce any mortality in rats. Throughout the 14-day observation period, no overt signs of acute toxicity were noted. Clinical parameters, including food and water consumption, remained within normal ranges, and no behavioral alterations, such as salivation, aggression, piloerection or abdominal writhing, were observed.

#### 2.4.3. Physical Observation and Mortality

No mortality or treatment-related toxic effects were observed in rats throughout the 14-day observation period. Detailed clinical monitoring revealed no abnormal physical or behavioral signs, including changes in skin and fur, eyes, mucous membranes, locomotor activity, tremors, salivation, diarrhea, sleep disturbances or coma.

#### 2.4.4. Acute Effect on Body Weight, Food and Water Consumption

Table 4 illustrates the alterations in average body weight, food intake and water consumption of female rats treated with methanol extracts of TL at doses of 300 and 2000 mg/kg on days 1, 7 and 14, relative to the control group. Throughout the 14-day period, rats receiving the TL extract showed a steady gain in body weight comparable to controls, indicating that the treatment did not induce acute toxic effects. Additionally, no significant variations were observed in their food and water consumption among all experimental groups.

#### 2.4.5. Hematological Parameters of Acute Toxicity

The effects of TL I and TL II extracts on the hematological parameters of female rats are presented in Table 5. After the administrations of TL extracts, a clinically significant statistical increase in red blood cell concentration was noted at the dosage of 300 mg/kg BW in comparison to the control group (*p* < 0.05). At the administered dose of 2000 mg/kg BW, hemoglobin levels showed a significant reduction relative to the control group (*p* < 0.01). A statistically significant reduction in both eosinophil count and percentage was observed at 300 and 2000 mg/kg BW doses in comparison to the control group (*p* < 0.01 and *p* < 0.001, respectively). Both the platelet count and the platelet larger cell ratio increased. The majority of hematological biomarkers are within normal limits when compared to the control group (*p* < 0.05) (Table 5).

#### 2.4.6. Effects of Plant Extracts on Biochemical Markers

The administration of TL I and TL II plant extracts led to statistically significant alterations in a wide range of biochemical markers associated with hepatic, renal, cardiac and lipid metabolic functions, indicating a potential systemic influence of these extracts on organ function and homeostasis. Specifically, hepatic enzyme activity, as reflected by alanine aminotransferase (ALT) and aspartate aminotransferase (AST) levels, demonstrated significant reductions in both treatment groups when compared to the control group (*p* < 0.01 and *p* < 0.05, respectively). These decreases may indicate a hepatoprotective effect or an overall improvement in liver function, as elevated transaminase levels are typically associated with hepatic injury or cellular leakage.

Conversely, a marked increase in alkaline phosphatase (ALP) levels was observed in the TL I-treated group (*p* < 0.01), which may reflect differential effects of this extract on hepatic or biliary physiology, or potentially point toward a response related to bone or intestinal ALP isoforms, warranting further investigation.

With respect to renal function, a significant reduction in serum uric acid levels was detected, alongside a dose-dependent decline in potassium concentrations compared to the control. These findings may indicate that the plant extracts influence renal clearance mechanisms or electrolyte homeostasis, which could have implications for their use in conditions involving hyperuricemia or electrolyte imbalance.

In the context of cardiac function, biomarkers such as lactate dehydrogenase (LDH) and ammonia (NH_3_) exhibited substantial decreases in both treatment groups. Creatine kinase (CK) level was also decreased in TL II group. The reduction in these markers may reflect an attenuation of cellular damage or membrane stabilization effects in cardiac tissues, supporting the hypothesis of a cardioprotective potential of the extracts.

Furthermore, the evaluation of lipid metabolism revealed a pronounced decrease in serum triglyceride levels, particularly in the TL II extract-treated group (*p* < 0.001). This finding suggests a beneficial modulatory effect on lipid homeostasis, possibly through mechanisms involving lipid synthesis, absorption or clearance.

Collectively, these biochemical outcomes suggest that both TL I and TL II plant extracts may exert protective, regulatory or therapeutic effects on multiple organ systems. The consistent alterations in key biochemical markers support the potential of these extracts to modulate physiological pathways relevant to hepatic, renal, cardiac and metabolic functions (Table 6). Further biochemical studies would be beneficial to elucidate the specific pathways involved in these observed effects.

#### 2.4.7. Gross Necropsy

The gross necropsy findings show no alterations in any of the investigated organs. The relative organ weight per 100 g of BW measured at the end of this study exhibited no significant difference compared to the control (Table 7).

#### 2.4.8. Histological Analyses

**Figure 5:**
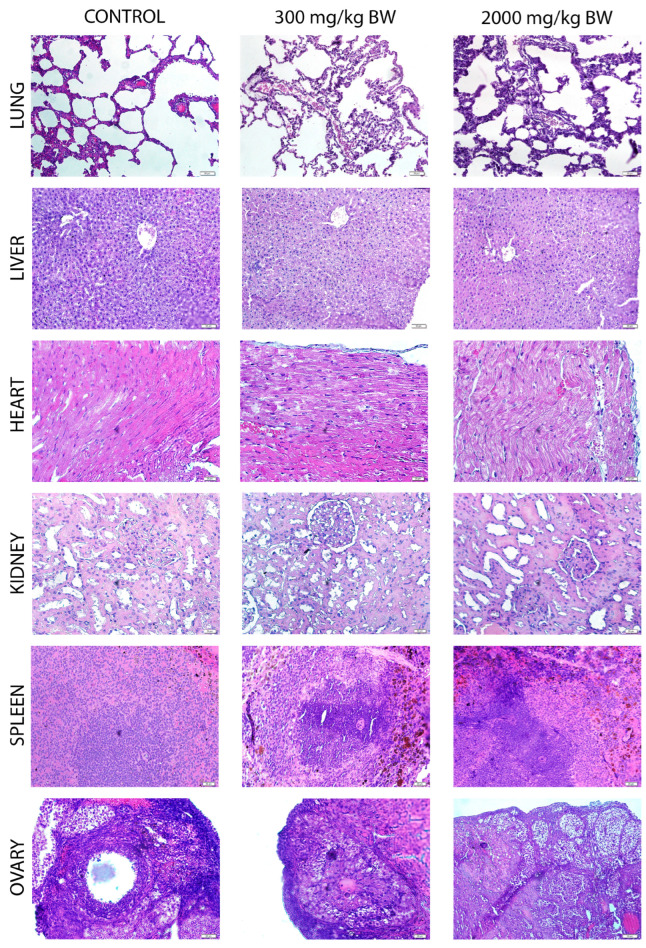
Representative lung, liver, heart, kidney, spleen and ovary micrographs are treated with 300 mg/kg BW (**middle** column) and 2000 mg/kg BW (**right** column) TL extract and control (**left** column). Magnification: ×200.

Histological evaluation of tissues, including lung, liver, heart, kidney, spleen, ovary, cerebrum and eye, revealed no significant structural differences among the control, 300 mg/kg BW and 2000 mg/kg BW experimental groups (Figure 5, Table 8). In all groups, the lung tissue demonstrated intact alveolar architecture, with no signs of edema or inflammation. Liver sections showed preserved hepatic lobular organization with normal hepatocyte morphology and sinusoidal structures. Similarly, cardiac tissue exhibited well-organized myocardial fibers without necrosis or inflammatory cell infiltration. Renal histology revealed normal glomeruli and tubules, with no evidence of interstitial damage or inflammatory cell infiltration. The spleen maintained its histological integrity, showing a well-defined red and white pulp structure. Ovarian sections were normal, with visible follicles and stromal tissue, indicating no histopathological abnormalities. Overall, the tissues from all groups exhibited preserved cellular and architectural integrity, suggesting no histopathological toxic effects related to the treatment.

The administration of TL extract at doses of 300 mg/kg BW and 2000 mg/kg BW did not induce significant histological alterations in the examined organs. The morphological assessment of tissue sections showed no discernible differences between the experimental and control groups, indicating that the extract does not exert histopathological alterations at the tested dose levels. This study supports the safety profile of the extract for acute exposure, with no observed histological damage. The semi-quantitative scores did not show statistical significance between the groups.

## 3. Discussion

The utilization of herbal medicines is increasing significantly globally. The worldwide endorsement of medicinal plants for disease treatment comes from their pharmacologically significant phytoconstituents, which are perceived as natural and consequently linked to minimal or no toxicity. The belief that herbal medications are safe and devoid of adverse effects is wrong. Various active compounds found in plants, which may elicit pharmacologically beneficial and/or detrimental effects [13]. Overuse of these medicinal plants may induce toxicity in tissues or organs. A toxicity assessment of the medicinal plant must be conducted to ensure its safe use as a plant-based medicine [14]. Therefore, it is important to assess the safety of the TL extract. Thus, in this study the safety profile of TL was assessed by acute oral toxicity following the OECD Guideline 420 procedure [15]. Also, cytotoxicity and genotoxicity were evaluated in NIH3T3 cells.

The chemical composition of TL extract provides important information about its safety profile. The high concentration of rosmarinic acid (87.37 µg/mg) detected in the extract is consistent with the previous studies on related *Thymus* species and ranks it as an important bioactive compound in this genus. When the relevant literature was examined, in another study, rosmarinic acid was analyzed as the major compound from *T. atlanticus* by the HPLC-DAD method. It was reported that the amount of rosmarinic acid varied in the range 2.74–69.38 mg/g from three different fractions obtained from the relevant study [16]. In another study, the *Thymus praecox* subsp. *grossheimii* var. *grossheimii* was found to contain 15.2 mg/g rosmarinic acid [17]. There are other studies in the literature indicating that *Thymus* species contain rosmarinic acid, and these data are consistent with the results of our study. In literature reviews, it was stated that rosmarinic acid has cytotoxic effects and inhibits colorectal cancer cells [18] and prostate cancer cells [19].

In our study, the methanol extract of TL was found to contain not only rosmarinic acid but also caffeic acid and apigenin. When compared with the findings from other studies on various Thymus species, our results show notable consistency. For instance, Sarfaraz et al. reported that methanol extracts of 11 different Thymus species contained rosmarinic acid as the predominant compound (32.3–139.5 mg/g), while the levels of caffeic acid ranged from 13.1 to 27.4 mg/g and apigenin from 9.3 to 56.7 mg/g [20]. The presence of caffeic acid and apigenin, albeit in lower amounts, is likely to contribute to the bioactivity of the extract. Overall, the safety profile combined with the high content of bioactive phenolic compounds makes TL extract a promising candidate for further preclinical and clinical studies. These findings will be particularly important for future studies investigating the pharmacological and medicinal administration of TL.

Although the development of dietary supplements and herbal medicines has become an active area of research and development, changes in the chemical content and biological activity of these therapeutic candidates may affect their efficacy and safety. Therefore, their toxicological profiles should be established. Therefore, cytotoxicity evaluations are crucial for determining the safety profile of plant extracts for potential therapeutic use. In the literature research, no studies were found on the cytotoxic profile of the TL plant, and this constitutes one of the unique aspects of this study. Regarding the impact of TL extract on cell viability, we observed a weak cytotoxic effect against the NIH3T3 cell line in a concentration-dependent manner. The research on *Thymus vulgaris* (thyme), one of the most studied species within its genus, has demonstrated selective cytotoxic effects on cancer cells, while preserving the viability of healthy cells. According to the review conducted by Afonso et al., the research on the cytotoxic effects of some Thymus phenolic-rich extracts on tumor cell lines showed that these extracts are effective in regulating tumor cell cycle, inducing programmed cell death and inhibiting angiogenesis. The obtained antioxidant and anti-inflammatory benefits support the potential use of Thymus extracts for the development of antitumor compounds [21]. Rosemary extracts exhibit antiproliferative effects against cancer cells, likely due to their polyphenol content, with caffeic and rosmarinic acids playing a central role [22]. In contrast, rosmarinic acid showed low cytotoxicity against non-tumorigenic cells [23]. The rich content of TL extract in terms of phenolic compounds, especially rosmarinic and caffeic acids, may explain its weak cytotoxic activity against non-cancerous NIH3T3 cells.

Growing evidence on polyphenols suggests that these compounds may offer considerable health benefits in the coming years. However, some polyphenols have been reported to exhibit dose-dependent toxic effects when consumed in large bolus doses [24]. This underscores the necessity of comprehensive toxicological assessments for polyphenol-enriched products. In this context, alongside evaluating the in vitro cytotoxicity of the TL extract, which is rich in polyphenols, a genotoxicity study was also conducted to further elucidate its toxicological profile. The genotoxic potential of TL extract on NIH3T3 cells was evaluated using both the comet assay and the MN test. The results from these analyses demonstrate that TL extract does not exert any significant genotoxic effects on NIH3T3 cells. In line with our findings, a previous study investigated the genotoxicity of Thymus vulgaris L. extract by assessing MN formation in various cell lines after 24 h of exposure. That study reported a protective effect of the extract against DNA damage. These results reflect that *Thymus vulgaris* extract may contribute to genomic stability and reduce mutagenicity, potentially due to the presence of thymol, its primary bioactive component [25]. Moreover, other studies have also reported similar protective or non-genotoxic effects associated with extracts from various Thymus species. For example, Calò et al. observed no DNA damage in keratinocytes treated with *Thymus vulgaris* extract, highlighting its potential as a genoprotective agent [26]. Collectively, these findings support the notion that Thymus species extracts, including TL, possess a low genotoxic risk.

Variations in BW have been utilized as a measure of the detrimental effects of pharmaceuticals and chemicals [27]. After conducting acute toxicity tests, no significant changes were observed in the general behavior and BW in the TL-treated groups as compared to the control group; it is suggested that with acute oral doses administered, TL had no effect on the normal growth of rats.

For the hematological parameter, EOS, EOS% and HGB were found to be significantly decreased as compared to the control rats. Eosinophils, bone marrow-derived granulocytes, are rare in healthy peripheral blood. Eosinophils produce and release granule proteins and pro-inflammatory mediators. Despite being present in all vertebrates, eosinophils’ function is still debated [28]. Eosinopenia has been associated with active infectious or inflammatory processes [29]. Occasional low eosinophil counts typically do not present a significant health risk, as other immune cells can compensate for the reduced eosinophil levels. Prolonged eosinopenia poses significant health risks and necessitates prompt intervention from a healthcare professional [30,31]. Our findings indicate that low eosinophilia does not present any risk.

Both the platelet count and the platelet larger cell ratio increased. The observed increase in platelets in rats treated with TL extract may be related to enhanced production within the bone marrow and secretion of thrombopoietin [32,33]. And, also, the primary regulator of platelet production may indicate that TL extract has hemostatic properties [34]. It is necessary to investigate whether chronic use diminishes platelet count by inhibiting production in the bone marrow or by causing platelets to aggregate and form clusters.

ALT, ALP and AST enzymes are abundantly present in the liver and are commonly used as biomarkers to assess hepatic function and detect liver disorders [35]. Damage to hepatocytes results in the release of these enzymes into the bloodstream, thereby elevating their serum levels and indicating hepatic injury. In particular, liver inflammation is often associated with a marked increase in transaminase activity [36]. Our results are consistent with earlier studies showing that TL methanolic extract exerts hepatoprotective effects, as evidenced by the reduction in AST and ALT enzyme levels [36,37]. Our results suggest that TL extract may play a role in mitigating liver cell damage. Supporting this, studies have shown that caffeic acid, a compound identified in TL extract, can prevent the elevation of serum liver enzymes and protect against hepatotoxicity induced by agents such as carbon tetrachloride (CCl_4_). This protective effect is thought to be mediated through enhanced antioxidant activity in hepatic tissue, which contributes to decreased levels of ALT and AST [38]. Moreover, flavonoids such as rosmarinic acid, also present in TL extract, are known for their potent antioxidant properties. These compounds help scavenge free radicals and reduce oxidative stress, thereby preventing cellular damage and contributing to the overall hepatoprotective profile of the extract [39,40].

Detailed biochemical assessments were conducted to evaluate the potential impact on renal function, emphasizing key markers, including BUN, creatinine, urea, uric acid, sodium and potassium levels. Xenobiotics undergo active filtration by the kidneys, which may result in their accumulation within the renal tubules. Elevated serum creatinine levels are commonly observed in instances of renal, cortical and/or glomerular injury [41]. In the present study, the levels of uric acid, phosphate and potassium were found to be significantly lower in the groups that were treated with TL extract in comparison to the control group. This study demonstrated no significant toxic effects on BUN or creatinine levels, thereby supporting the assertion that TL extract does not negatively impact renal function. The histological examination of the kidneys in both the treated and control groups corroborated the biochemical findings, revealing no significant morphological differences and confirming the absence of renal toxicity of the plant following acute exposure.

Cardiac toxicity markers (LDH, CK, CO_2_, NH_3_, lipase and amylase) were evaluated in serum samples. TL-treated groups showed a significant decrease in LDH, CK enzymes and NH_3_ level versus the control group. Cardiovascular diseases are associated with blood triglyceride levels [42]. Triglyceride levels were significantly lower in all treatment groups compared to control groups. Intrahepatic fat and visceral fat have been shown to be independently associated with metabolic dysfunction [42,43]. As a result of our study, no evidence of steatosis was found in the liver histopathology results in the treated groups, similar to the control group.

Despite these promising findings, this study has several limitations that should be considered. First, this study only evaluated acute oral toxicity and therefore does not reflect the potential effects resulting from long-term or repeated use of the TL extract. Subacute, subchronic and chronic toxicity studies are necessary to fully determine the safety profile of TL extract in therapeutic contexts. Second, although we performed extensive hematological, biochemical and histological evaluations, this study did not include molecular analyses that could further elucidate the mechanistic aspects of its biological activity, such as oxidative stress markers or proinflammatory cytokines. Thirdly, the results obtained are specific to the methanol extract of aerial parts and may not be generalizable to other types of extracts or preparation methods. Finally, we believe that this dual approach using a non-tumorigenic fibroblast cell line along with in vivo acute toxicity assessment provides a meaningful and ethically balanced preliminary safety evaluation of the plant extract. However, we acknowledge that the safety profile should be further expanded by including a broader range of human non-tumorigenic cell types, such as epithelial primary cultures, NeHepLxHT, MCF-10A and L-929 in future studies. These limitations emphasize the need for further preclinical studies to explore the full toxicological spectrum and therapeutic potential of *T. longicaulis.*

## 4. Materials and Methods

### 4.1. Data Collection and Identification of Plant Sample

The plant samples of TL were collected from Taşköprü (Kastamonu), Türkiye, and taxonomically described using the *Flora of Turkey and the East Aegean Islands* [6] by Dr. Ismail Senkardes. Additionally, the scientific nomenclature of the plant taxa was verified and revised in accordance with the latest standards provided by the World Flora Online database [44]. A voucher specimen of the aerial parts of TL was stored at the Herbarium of the Faculty of Pharmacy at Marmara University (voucher number: MARE-19081).

### 4.2. Preparation of Plant Extracts

The plant samples were subsequently air-dried in a shaded environment and ground. A total of 100 g dry powder of TL was extracted using a maceration process with methanol (100%) (methanol was preferred for the extraction of polar components from the plant). The extraction process was continued until the solvent became colorless. The obtained liquid extract was filtered through filter paper. Subsequently, the methanolic solvent was removed under reduced pressure at 45 °C using a rotary evaporator (Heidolph, Schwabach, Germany) until complete dryness was achieved and the obtained dry extracts were stored in a refrigerator at +4 °C [45]. As a result of this study, a solid-form extract was obtained and the yield of the obtained extract was found to be 2.35%.

### 4.3. Analysis of Phenolic Compounds

In this study, the Agilent HPLC system 1260 Infinity II (Agilent technology, Santa Clara, CA, USA) was utilized to analyze the phenolics in methanol extracts from TL. The parameters of the HPLC system (mobile phase, temperature, flow rate, etc.) were optimized for the analysis of phenolic compounds in the extracts using the HPLC-DAD method. For this purpose, first of all, gradient elution methods were performed with different mobile phases and different compositions using an ACE 5 C18 250 × 4 mm column. The best eluent was selected using mobile phases containing methanol and acetonitrile for phase B and different amounts of acetic acid, formic acid and phosphoric acid for phase A. It was decided that the mobile phase containing acetonitrile for phase B and 0.1% formic acid for phase A provided the best separation. Therefore, an experiment was also performed with a Novapak C18- 4 µm × 3.9 × 150 mm Waters column under the same conditions, but since the peak symmetries were more suitable for the desired reference range with the 25 cm ACE column and therefore the peak resolution was better, the 15 cm column was not found suitable for the analysis. The elution gradients were as follows: 0–15% B (0–5 min); 15–25% B (5–10 min); 25–35% B (10–15 min); 35–40% B (15–20 min); 40–65% B (20–25 min); 65–15% B (25–32 min); 15–0% B (32–35 min). A value of 330 nm was chosen as the optimal wavelength for diode-array detection (DAD). The metabolites were identified on the basis of their DAD spectra and retention times. Preliminary tests were performed at 25, 30 and 35 °C for the HPLC column temperature and it was decided that 30 °C was the optimum temperature. In addition, flow rate tests were conducted at 0.2–0.5 and 0.7 mL/min and it was decided to work at a flow rate of 0.5 mL/min. To analyze phytochemical substances quantitatively, a calibration curve was created by injecting known amounts of several standards with different concentrations under the above-mentioned optimum condition. Each injection consisted of three replicates. The results are presented as µg/mg of the extract.

### 4.4. Cytotoxicity Assay

The cytotoxic potential of TL extract was assessed using the mouse embryonic fibroblast cell line NIH3T3 (ATCC-CRL-1658) (American Type Culture Collection, Rockville, MD) at passage 34. Cells were cultured in DMEM/F12 medium supplemented with 10% fetal bovine serum (FBS), 1% L-glutamine and penicillin/streptomycin (Gibco, NY, USA) at 37 °C in a humidified incubator with 5% CO_2_. Cytotoxicity was evaluated via the MTT assay [46]. Briefly, cells were seeded at a density of 1 × 10^4^ cells per well in 96-well plates and allowed to adhere overnight. Subsequently, cells were exposed to varying concentrations of TL extract (10, 20, 50, 100 and 200 µg/mL) for 24 h. Following treatment, MTT reagent was added to each well at a final concentration of 5 mg/mL and incubated for 4 h. The culture medium was then discarded, and 100 µL of SDS buffer was added to dissolve the formazan crystals. Absorbance was measured at 570 nm and 630 nm using a microplate reader (Biotek, Winooski, VT, USA). All experiments were conducted in duplicate, with each treatment performed in triplicate. Cell viability (%) was calculated using the following formula:

% Cell Viability = [(Mean OD of treated cells)/(Mean OD of control cells)] × 100

### 4.5. In Vitro Genotoxicity Evaluation

#### 4.5.1. Comet Assay

The comet technique was applied to investigate the possible genotoxic effect of TL extracts on NIH3T3 cells. The cells were seeded into 6-well plates at a concentration of 1 × 10^5^ cells per well and subsequently treated with TL extracts at final concentrations of 50, 100 and 200 µg/mL. Following a 24 h incubation period, cells were harvested by trypsinization and resuspended in culture medium for use in the comet assay. The assay was conducted under alkaline conditions following the protocol described by Singh et al., with minor modifications [47]. Cells were suspended in low-melting agarose and gently spread onto slides previously coated with high-melting-point agarose. Slides were solidified at 4 °C for 15 min and subsequently transferred into a lysing solution, where they were incubated for 1 h at 4 °C. Following the lysis procedure, the slides were rinsed with ice-cold distilled water and subsequently placed in a horizontal electrophoresis chamber containing a freshly prepared alkaline buffer (300 mM NaOH, 1 mM EDTA; pH > 13). The slides were incubated in the buffer for 20 min at 4 °C to facilitate DNA unwinding prior to electrophoresis. Electrophoresis was then carried out under alkaline conditions for 20 min at a constant current of 300 mA and a voltage of 15 V. At the end of electrophoresis, slides were rinsed with precooled distilled water and were neutralized to eliminate residual alkali in Tris buffer (0.4 M, pH 7.5). Slides were stained with ethidium bromide, and image analysis was performed at 400× magnification using a fluorescent microscope (Olympus, BX51, Tokyo, Japan). The percentage of DNA in the tail (%DNA_T_) was used as an indicator of DNA damage, assessed by scoring 100 cells across two duplicate sample slides, with 50 randomly selected cells evaluated per slide, utilizing BAB Bs200Pro image analysis software (BAB Ltd., Ankara, Turkey). The evaluation was performed by comparing the control group and positive control (50 μM H_2_O_2_ concentration, known to cause DNA damage, was used as the positive control [48]).

All data are expressed as the mean and standard deviation of the mean obtained by repeating each test at least three times.

#### 4.5.2. MN Test

The MN test was carried out according to Fenech [49,50]. NIH3T3 cells were seeded into each well of 6-well sterile plates at 2 × 10^5^. The cells were incubated for 24 h in an incubator. At the 24th hour, TL extract was applied to NIH3T3 cells at concentrations of 50–100–200 µg/mL. Mitomycin-C (MMC) was applied at a final concentration of 0.2 µg/mL as a positive control [51] . The same volume of medium was used for the negative control. After 44 h of incubation, 6 µg/mL Cytochalasin B was added to block cytokinesis, resulting in the formation of binucleated (BN) cells. The cells were incubated for 28 h and cells were taken out of the cultures by trypsinization. After the cell-washing process was completed, 0.075 M KCl hypotonic solution at 4 °C was added and then kept in the refrigerator for 5 min. Subsequently, cells were centrifuged again and fixed three times using a cold methanol/glacial acetic acid solution (3:1). During the final fixation step, 1% formaldehyde was added to preserve the cytoplasmic structure. Prepared slides were made by dropping the cell suspension followed by air-drying. The slides were then stained with 5% Giemsa solution (pH 6.8) in Sorensen buffer for 15 min, rinsed with distilled water and allowed to dry at room temperature. The analysis was performed using a light microscope (Olympus, BX51, Tokyo, Japan) with a 400× magnification. The samples were coded and scored blindly by the same analyst. Three slides per dose were prepared, in which 3000 binucleated cells were collectively analyzed for the presence of MN.

Micronuclei were recognized as distinct entities independent from the two primary nuclei, morphologically analogous to, although smaller than, them. Mean values and standard errors of the mean (SEM) for MN frequency were computed using the comprehensive data collected across all experiments for each cell line. The frequency of each parameter is expressed as MN/cell.

Cell proliferation in vitro was evaluated by counting the frequencies of mono-, bi-, tri- and tetra-nuclear cells in the first 500 cells counted on each slide for each concentration, and the results are presented as the cytokinesis-block proliferation index (CBPI)

CBPI = (M1 + 2M2 + 3M3 + 4M4)/N,

where M1–M4 represent the number of cells with 1–4 nuclei, and N is the total number of viable cells scored (excluding necrotic and apoptotic cells).

### 4.6. Animals and Experimental Design

A total of 20 female Wistar rats (250 ± 20 g) were obtained from the Experimental Animal Implementation and Research Center (DEHAMER) of Marmara University. The study protocol was approved by the Marmara University Animal Experiments Local Ethics Committee (Approval No: 46.mar.2022). All procedures were conducted following ethical principles for animal research and the Guide for the Care and Use of Laboratory Animals. Throughout the study, the environment was maintained according to the following guideline conditions: 22–26 °C, 55 ± 15% relative humidity and a 12 h light/dark cycle. The rats were numbered for identification and recording purposes and were provided access to unlimited food and water. Before the beginning of the experiment, they were acclimatized for one week.

### 4.7. Acute Toxicity

The acute toxicity tests were conducted in accordance with the OECD Guideline for the Testing of Chemicals, Test No. 420. TL extract was prepared by dissolving in drinking water. The fixed doses specified in the guideline for the preliminary study are included (5, 50, 300 and 2000 mg/kg body weight (BW)), along with 300 mg/kg, which was selected as the starting dose. The preliminary studies were conducted with two animals per group. Rats received a single oral dose of 300 mg/kg TL extract via the intragastric route, and the rats were evaluated in view of mortality and toxicity. If no toxic effects were observed, the same procedures were applied in the other group for the dose of 2000 mg/kg BW. Following a two-day observation, the main study was initiated, based on the absence of toxicity and mortality [52]. In the main study, there were three groups (five rats per group):

Control: Vehicle control.

- TL I: TL extract was administered intragastrically at 300 mg/kg BW.
- TL II: TL extract was administered intragastrically at 2000 mg/kg BW.

In the main study, observations were made for 14 days for signs of mortality and toxicity. Rats were observed for clinical signs such as pain, agitation, groaning, skin irritation, stool consistency, eyes, behavior patterns and coma. Food–water consumption and weight measurements were performed on days 1, 7 and 14 [52,53,54]. The animals at the end of the experiment were fasted overnight (for 12 h) before receiving anesthesia and sacrificed following intracardiac blood collection.

### 4.8. Hematological and Biochemical Analyses

Fresh blood samples were collected via intracardiac blood collection using a sterile syringe as much as 1–3 mL for the analysis of the hematological and biochemical parameters. For the hematological analysis, blood counts were performed using BD Vacutainer blood collection tubes containing K2 EDTA. The parameters examined included red blood cells (RBCs), platelets (PLTs), hematocrit (HCT), white blood cells (WBCs), hemoglobin (HGB), immature granulocytes (IMGs), monocytes (MONs), percentage of monocytes (%MON), lymphocyte (LYM), percentage of lymphocytes (%LYM), neutrophils (NEUs), percentage of neutrophils (%NEU), eosinophils (EOSs), percentage of eosinophils (%EOS), basophils (BASs), percentage of basophils (%BAS), percentage of immature granulocytes (%IMG), mean corpuscular volume (MCV), mean corpuscular hemoglobin (MCH), mean corpuscular hemoglobin concentration (MCHC), coefficient of variation in red cell distribution width (RDV-CV), mean platelet volume (MPV), platelet distribution width (PDW), procalcitonin (PCT), platelet larger cell count (PLCC) and platelet larger cell ratio (PLCR). Hematological parameters, including white blood cell count, red blood cell count, hemoglobin concentration, hematocrit and platelet count, were analyzed using an Automated Hematology Analyzer (Mindray BC-6200, Shenzhen Mindray Bio-Medical Electronics Co., Ltd., Shenzhen, China). This analyzer utilizes flow cytometry, laser scattering and impedance-based technologies to deliver accurate and reproducible complete blood count (CBC) results.

For the biochemical analyses, 1 mL of blood was transferred to a tube containing a separating gel and was subjected to centrifugation for a period of 10 min at a speed of 3000 rpm until serum was produced. The parameters examined included alanine transaminase (ALT), urea, alkaline phosphatase (ALP), blood urea nitrogen (BUN), uric acid (UA), aspartate aminotransferase (AST), Mg, phosphate, Ca, Na, K, Cl, lactate dehydrogenase (LDH), creatine kinase (CK), NH_3_, lipase, amylase, total cholesterol, glucose and triglyceride [55]. These biochemistry parameters were measured using a Clinical Chemistry Auto-analyzer (Roche cobas^®^ 6000 c501 modular analyzer, Roche Diagnostics GmbH, Mannheim, Germany). This automated system provides high-throughput and precise quantifications of serum analytes, ensuring consistency across all samples.

### 4.9. Observation of Organs and Histological Examination

The excised lung, liver, heart, kidney, spleen, ovary, cerebrum and eye were cleaned, and their color and consistency were evaluated. The weights of each organ were measured, then their relative weights were calculated using the formula below [56]:

$$Relative\;organ\;weight\;(\%)=\frac{Organ\;weight}{Body\;weight}\times 100$$

Lung, liver, heart, kidney, spleen and ovary tissues from the experimental groups were carefully fixed in 10% formaldehyde to preserve cellular and tissue architecture. Following fixation, the samples underwent standard histological processing using an automated tissue processor (Leica TP1020, Nussloch, Germany). The tissue processing procedure involved stepwise dehydration through graded concentrations of ethanol, followed by xylene-mediated clearing and subsequent infiltration with molten paraffin wax. The processed tissues were then embedded in paraffin using the Leica EG1150H + C embedding system, ensuring consistent orientation for accurate sectioning. Paraffin-embedded tissue blocks were sectioned at a thickness of 5 µm using a rotary microtome (Leica RM2125RT), under standardized conditions to preserve histomorphological integrity and to minimize sectioning-related artifacts. These sections were mounted onto glass slides and dried to enhance adhesion. Histological analysis was performed following hematoxylin and eosin (H&E) staining of the paraffin sections, allowing for a detailed assessment of tissue morphology and pathological alterations. Hematoxylin provided a deep-blue-to-purple stain of the nuclei, enabling the clear visualization of nuclear structures, while eosin counterstained the cytoplasm and extracellular matrix in shades of pink, emphasizing overall tissue morphology.

For the semi-quantitative histopathological evaluation of organ damage, tissue sections were examined under a light microscope, and histopathological changes were scored based on established grading scales from previous studies. Five-to-ten fields (20× magnification) were randomly selected from the tissue sections and scored by an independent, blinded histologist to ensure objectivity.

Liver sections were assessed for the severity of hepatocellular vacuolization, hepatocellular degeneration with pyknotic nuclei, sinusoidal dilatation, fibrosis, neutrophil infiltration and Kupffer cell activation, following the previously published methods on a scale from 0 to 3 (0 = none, 1 = mild, 2 = moderate and 3 = severe) [57].

Ovarian tissue damage was assessed based on the presence and severity of interstitial edema, follicular cell degeneration (granulosa cells), hemorrhage, polymorphonuclear leukocyte (PMN) infiltration and vascular dilatation. Damage was graded on a scale from 0 to 3 (0 = none, 1 = mild, 2 = moderate and 3 = severe) [58].

Morphological damage in the kidney tissue was quantified based on the following criteria: tubular injury, edema, congestion, hyalinization, tubular degeneration and tubular dilatation scored using a semi-quantitative scale: none = 0, <25% = 1, 25–50% = 2, 50–75% = 3 and >75% = 4, as previously described [59].

The histopathological evaluation of cardiac tissue was performed based on the extent of fibrosis (interstitial and replacement) and severity of cardiomyocyte alterations, including nuclear atypia, disarrangement, sarcoplasmic rarefaction, cardiomyocyte tapering and cytoplasmic vacuolation. The scoring system was graded as follows: 0, absent; 1, <5%; 2, 5–10%; 3, 10–20% and 4, >20% of the myocardium [60].

Lung tissue sections were analyzed according to the following criteria: intra-alveolar hemorrhage, alveolar disruption, leukocyte infiltration, alveolar wall thickening and intra-alveolar edema [61]. The changes were scored semi-quantitatively on a scale from 0 to 3 (0 = none; 1 = mild; 2 = moderate; 3 = severe) [62].

For the semi-quantitative evaluation of spleen tissue damage, structural abnormalities were graded from 0 (normal structure) to 3 (severe pathological changes). Histopathological assessment included the evaluation of splenic architecture, lymphoid follicle integrity, white pulp organization and red pulp alterations [63].

A histopathological evaluation of the cerebral cortex was performed using a semi-quantitative scoring system consisting of four categories, ranging from 0 (normal cortical structure) to 3 (severe disruption of cortical lamination and significant atrophy/neuronal loss). This scoring system allows for the assessment of cortical structural integrity and has been utilized in studies examining cortical alterations in various neurological conditions [64].

For a histopathological evaluation of eye tissues, a semi-quantitative scoring system was used to assess the severity of tissue damage. The scoring criteria included the evaluation of epithelial erosion, stromal edema, inflammatory cell infiltration and endothelial cell loss. Each parameter was graded on a scale from 0 to 3 (0 = none, 1 = mild, 2 = moderate and 3 = severe) [65].

### 4.10. Statistical Analysis

Data are presented as mean values with standard deviations (mean ± SD), and statistical analyses were performed using GraphPad Prism version 9. The data were subjected to one-way analysis of variance (ANOVA), followed by the Bonferroni post-test. In order to evaluate the results of the comet and MN assay, the Mann–Whitney U test was used to compare paired groups that did not conform to the normal distribution, and the Kruskal–Wallis variance analysis was used to compare more than two groups. A *p*-value of less than 0.05 was considered to be statistically significant.

## 5. Conclusions

In conclusion, this study provides the first comprehensive evaluation of acute oral toxicity, cytotoxicity and genotoxicity of TL methanol extract. The findings indicate that the TL extract was well-tolerated at the tested doses and no significant toxic effects were observed on hematological, biochemical or histopathological parameters. In this context, the acute oral toxicity of the TL extract was determined as grade 5 according to the OECD 420 guideline. The weak cytotoxic activity observed in NIH3T3 cells, together with the absence of genotoxic effects, suggests a favorable safety profile. The high content of phenolic compounds, especially rosmarinic and caffeic acids, may contribute to both the observed bioactivities and the antioxidant potential of the extract. However, considering the limitations of this study—particularly its focus on acute toxicity—further studies including subchronic and chronic toxicity models are required. Overall, these results support the potential of *T. longicaulis* subsp. *chaubardii* as a safe candidate for further pharmacological and therapeutic research.

## Figures and Tables

**Table 1:** Values of chemical compounds of TL extracts.

Component	Average (µg/mg of the Extract)
Caffeic acid	31.22 ± 1.92
Rosmarinic acid	87.37 ± 5.39
Apigenin	1.12 ± 0.18

**Table 2:** The genotoxic effects of TL extracts on NIH3T3 cells, assessed using the comet technique.

		DNA_T_ (%)
Control		20.46 ± 0.40
Positive Control (H_2_O_2_)		66.30 ± 0.92 ***
*Thymus longicaulis* subsp. *chaubardii*	50 µg/mL	21.87 ± 0.70 ^###^
	100 µg/mL	26.54 ± 0.83 ***^,###^
	200 µg/mL	27.28 ± 0.78 ***^,###^

Data are shown as mean ± standard deviation. *** *p* < 0.001 compared to control group; ^###^ *p* < 0.001 compared to positive control group. DNA_T_ (%): Percentage of DNA in tail.

**Table 3:** Assessment of genotoxic effects of TL extracts on NIH3T3 cells via the MN test.

		BN	Total Number of MNi		MN/Cell ± SE	CBPI ± SE
			(1)	(2)		
Control		3000	6	-	0.002 ± 0.0001 ^###^	1.65 ± 0.01 ^#^
*Thymus longicaulis* subsp. *chaubardii*	50 µg/mL	3000	17	1	0.0063 ± 0.00006 ^###^	1.63 ± 0.01
	100 µg/mL	3000	28	4	0.012 ± 0.0002 **^,###^	1.60 ± 0.005
	200 µg/mL	3000	39	4	0.0156 ± 0.0002 ***^,###^	1.59 ± 0.01
Mitomycin C		3000	180	16	0.0706 ± 0.0004 ***	1.33 ± 0.006

Data are shown as mean ± standard error. ** *p* < 0.01 and *** *p* < 0.001 compared to control group; ^#^ *p* < 0.05 and ^###^ *p* < 0.001 compared to positive control group. (1) Cell with one MN; (2) cell with two MNi; BN:binucleate cells; MNi: micronuclei; MN: micronucleus; CBPI: cytokinesis-blocked proliferation index.

**Table 4:** Body weight (g), food consumption (g) and water intake (mL) by control and rats treated with TL extract recorded during the acute toxicity study.

Groups	Body Weight (g)			Food Consumption (g)			Water Intake (mL)		
	Day 1	Day 7	Day 14	Day 1	Day 7	Day 14	Day 1	Day 7	Day 14
Control	225.03 ± 6.45	240.72 ± 5.35	248.90 ± 6.48	25.40 ± 1.08	26.01 ± 0.17	25.84 ± 2.10	14.40 ± 1.20	15.80 ± 0.83	15.74 ± 1.43
TL I	238.86 ± 5.87	250.94 ± 7.45	250.24 ± 10.35	24.70 ± 2.58	25.90 ± 1.39	23.74 ± 3.39	16.35 ± 2.49	15.47 ± 1.59	14.87 ± 2.69
TL II	220.48 ± 9.34	230.38 ± 8.43	233.56 ± 9.45	26.53 ± 0.98	25.76 ± 1.32	27.45 ± 1.15	16.76 ± 3.56	17.54 ± 2.54	14.65 ± 2.54

TL I: TL extract was administered intragastrically at 300 mg/kg BW; TL II: TL extract was administered intragastrically at 2000 mg/kg BW. Values are represented as mean + SD (*n* = 5).

**Table 5:** Hematological values of control and rats treated with TL extract measured at the end of the acute toxicity study. Hematological parameters were assessed using an Automated Hematology Analyzer (Mindray BC-6200).

Parameters	Control	TL I	TL II
WBC (10^3^/µL)	5.20 ± 1.88	8.03 ± 3.31	7.58 ± 2.19
NEU (10^3^/µL)	0.91 ± 0.15	1.36 ± 0.63	1.97 ± 0.59
LYM (10^3^/µL)	3.74 ± 1.65	6.13 ± 2.48	5.03 ± 1.38
MON (10^3^/µL)	0.20 ± 0.05	0.36 ± 0.17	0.45 ± 0.19
EOS (10^3^/µL)	0.34 ± 0.10	0.18 ± 0.10 **	0.12 ± 0.03 ***
BAS (10^3^/µL)	0.01 ± 0.01	0.01 ± 0.01	0.003 ± 0.004
IMG (10^3^/µL)	0.02 ± 0.01	0.02 ± 0.01	0.56 ± 0.36
NEU%	18.58 ± 3.62	16.90 ± 3.50	25.86 ± 0.43
LYM%	70.35 ± 6.04	76.53 ± 3.91	67.06 ± 1.63
MON%	3.95 ± 0.77	4.45 ± 0.59	5.43 ± 1.20
EOS%	6.95 ± 2.35	2.05 ± 0.39 ***	1.57 ± 0.04 ***
BAS%	0.22 ± 0.03	0.09 ± 0.01	0.06 ± 0.04
IMG%	0.43 ± 0.11	0.20 ± 0.07	0.56 ± 0.36
RBC (10^6^/µL)	7.69 ± 0.45	8.22 ± 0.32 *	7.38 ± 0.07
HGB (g/dL)	14.13 ± 0.44	14.58 ± 0.44	12.98 ± 0.27 **
HCT (%)	41.60 ± 2.73	41.95 ± 1.52	39.22 ± 0.50
MCV (fL/cell)	54.10 ± 0.66	51.07 ± 1.58	53.06 ± 1.06
MCH (pg/cell)	18.42 ± 0.60	17.75 ± 0.46	17.60 ± 0.50
MCHC (g/dL)	34.08 ± 1.27	34.73 ± 0.41	33.16 ± 0.27
RDW-CV (%)	12.38 ± 0.20	12.70 ± 0.58	14.03 ± 0.11
RDW-SD (fL)	26.80 ± 0.76	25.98 ± 0.73	29.78 ± 0.75
PLT (10^3^/µL)	304.8 ± 220.6	743.8 ± 83.78 ***	896.3 ± 14.51 ***
MPV (fL)	8.88 ± 0.70	8.00 ± 0.25	7.63 ± 0.20 **
PDW (fL)	15.98 ± 0.53	15.45 ± 0.05	15.23 ± 0.04
PCT (ng/mL)	0.25 ± 0.16	0.59 ± 0.07	273.3 ± 373.3
P-LCC (10^9^/L)	50.50 ± 17.36	105.5 ± 16.41 **	102.3 ± 10.16 **
P-LCR (%)	21.15 ± 6.51	14.23 ± 1.72 **	11.43 ± 1.00 ***

NEU = Neutrophil; MON = Monocyte; LYM = Lymphocyte; EOS = Eosinophil; BAS = Basophil; IMG = immature granulocyte; NEU% = Percentage of neutrophils; MON% = Percentage of monocytes; HGB = Hemoglobin; EOS% = Percentage of eosinophils; WBC = White blood cells; PLT = Platelet; MCV = Mean corpuscular volume; BAS% = Percentage of basophils; LYM% = Percentage of lymphocytes; IMG% = Percentage of immature granulocytes; P-LCC = Platelet larger cell count; RBC = Red blood cell; HCT = Hematocrit; MCH = Mean corpuscular hemoglobin; RDW-SD = Standard deviation in red cell distribution width; MCHC = Mean corpuscular hemoglobin concentration; RDW-CV = Coefficient of variation in red cell distribution width; MPV = Mean platelet volume; PDW = Platelet distribution width; P-LCR = Platelet larger cell ratio; PCT = Procalcitonin; pg (pictogram). TL I: TL extract was administered intragastrically at 300 mg/kg BW; TL II: TL extract was administered intragastrically at 2000 mg/kg BW. Values are represented as mean + SD (*n* = 5). * *p* < 0.05, ** *p* < 0.01, and *** *p* < 0.001 indicate significant changes in comparison with the normal control.

**Table 6:** Clinical biochemistry values of control and rats treated with TL extract measured at the end of the acute toxicity study. Clinical biochemistry data were measured by a Clinical Chemistry Auto-analyzer (Roche cobas^®^6000 c501 modular analyzer).

Parameters	Control	TL I	TL II
**Liver profile**			
ALT (U/L)	143.2 ± 91.13	48.20 ± 6.18 **	54.20 ± 2.05 **
AST (U/L)	342.6 ± 223.0	158.2 ± 37.08 *	145.0 ± 32.46 *
ALP (U/L)	85.80 ± 34.76	128.6 ± 14.45 *	81.80 ± 21.65
Total protein (g/L)	73.40 ± 29.48	73.70 ± 1.75	69.08 ± 1.21
Albumin (g/L)	49.60 ± 2.91	51.72 ± 1.42	46.38 ± 1.29
**Renal profile**			
Urea (mg/dL)	44.74 ± 3.12	48.60 ± 1.98	42.56 ± 3.65
BUN (mg/dL)	20.91 ± 1.46	22.54 ± 1.01	19.64 ± 1.78
Creatine (mg/dL)	0.41 ± 0.04	0.37 ± 0.03	0.37 ± 0.01
UA (mg/dL)	2.12 ± 0.99	1.15 ± 0.25 *	1.40 ± 0.12
Mg	2.76 ± 0.27	2.54 ± 0.04	2.35 ± 0.08
Phosphate	5.68 ± 1.01	3.98 ± 0.23 **	4.63 ± 0.21
Ca (mg/dL)	11.01 ± 0.67	10.84 ± 0.24	10.99 ± 0.19
Na	145.2 ± 3.70	143.5 ± 2.60	144.0 ± 0.00
K	6.72 ± 1.58	5.29 ± 0.17 *	4.80 ± 0.04 **
Cl	105.3 ± 2.13	103.6 ± 0.74	104.4 ± 0.46
**Cardiac profile**			
LDH (U/L)	2156 ± 921.9	1200 ± 203.8 *	1120 ± 376.4 *
CK (U/L)	1016 ± 109.29	1104 ± 283.3	629.8 ± 183.5 *
CO_2_	12.42 ± 4.16	15.26 ± 1.35	15.24 ± 1.37
NH_3_	318.4 ± 139.8	197.3 ± 20.60 *	183.5 ± 8.77 *
Lipase	8.40 ± 52.41	8.40 ± 0.07	8.77 ± 0.65
Amylase	2266 ± 275.0	2271 ± 146.2	1842 ± 177.8
**Lipid profile**			
Total cholesterol (mmol/L)	72.38 ± 6.624	86.76 ± 7.36 *	78.18 ± 4.79
Glucose (mmol/L)	157.2 ± 30.28	141.0 ± 10.32	146.0 ± 6.16
Triglyceride (mmol/L)	303.4 ± 41.08	208.4 ± 101.9	132.4 ± 15.75 ***

ALT = Alanine transaminase; AST = Aspartate aminotransferase; ALP = Alkaline phosphatase; BUN = Blood urea nitrogen; UA = Uric acid; LDH = Lactate dehydrogenase; CK = Creatine kinase; CO_2_ = Carbondioxide; NH_3_ = Ammonia. TL I: TL extract was administered intragastrically at 300 mg/kg BW; TL II: TL extract was administered intragastrically at 2000 mg/kg BW. Values are represented as mean + SD (*n* = 5). * *p* < 0.05, ** *p* < 0.01, and *** *p* < 0.001 indicate significant changes in comparison with the normal control.

**Table 7:** Values of control and rats treated with TL extract measured by the acute toxicity study. The relative organ weight per 100 g BW recorded at the end of the study.

	Control	TL I	TL II
Lung	0.57 ± 0.14	0.53 ± 0.18	0.55 ± 0.14
Liver	4.15 ± 1.26	3.99 ± 0.98	4.08 ± 1.26
Heart	0.47 ± 0.06	0.46 ± 0.04	0.51 ± 0.06
Spleen	0.24 ± 0.05	0.25 ± 0.13	0.26 ± 0.15
Kidney Left	0.40 ± 0.05	0.39 ± 0.04	0.40 ± 0.25
Kidney Right	0.39 ± 0.04	0.39 ± 0.25	0.38 ± 0.64
Ovary Left	0.03 ± 0.01	0.03 ± 0.01	0.03 ± 0.01
Ovary Right	0.03 ± 0.01	0.03 ± 0.01	0.03 ± 0.01

TL I: TL extract was administered intragastrically at 300 mg/kg BW; TL II: TL extract was administered intragastrically at 2000 mg/kg BW. Values are represented as mean + SD (*n* = 5).

**Table 8:** Organ damage scoring in all tissues.

	Control (Mean ± SD)	TL Extract (300 mg/kg BW) (Mean ± SD)	TL Extract (2000 mg/kg BW) (Mean ± SD)
Lung	0.5 ± 0.41	0.75 ± 0.50	1.0 ± 0.63
Liver	0.4 ± 0.52	1.2 ± 0.41	1.0 ± 0.63
Heart	0.2 ± 0.41	0.5 ± 0.55	1.2 ± 0.41
Kidney	1.0 ± 0.63	1.5 ± 0.55	2.0 ± 0.63
Spleen	0.0 ± 0.00	0.7 ± 0.52	1.0 ± 0.63
Ovary	0.7 ± 0.52	1.0 ± 0.63	1.5 ± 0.55
Cerebrum	0.5 ± 0.55	2.0 ± 0.63	1.0 ± 0.63
Eye	0.5 ± 0.55	1.0 ± 0.63	1.5 ± 0.55

Semi-quantitative scoring was performed by an independent, blinded histologist using 5–10 randomly selected fields per tissue section at 20× magnification. Scoring criteria varied by organ and were derived from previously published methods: Lung, Liver, Ovary and Eye: 0 = none, 1 = mild, 2 = moderate and 3 = severe. Kidney: 0 = none, 1 = <25%, 2 = 25–50%, 3 = 50–75% and 4 = >75% damage. Heart: 0 = absent, 1 = <5%, 2 = 5–10%, 3 = 10–20% and 4 = >20% of myocardium affected. Spleen and cerebrum: 0 = normal, 1 = mild, 2 = moderate and 3 = severe disruption.

## Data Availability

The raw data supporting the conclusions of this article will be made available by the authors on request. The data are not publicly available due to its involvement in ongoing related research.

## Abbreviations

The following abbreviations are used in this manuscript:

%BAS	Percentage of basophils
%EOS	Percentage of eosinophils
%IMG	Percentage of immature granulocytes
%LYM	Percentage of lymphocytes
%MON	Percentage of monocytes
%NEU	Percentage of neutrophils
ALP	Alkaline phosphatase
ALT	Alanine transaminase
ANOVA	One-way analysis of variance
AST	Aspartate aminotransferase
BASs	Basophils
BHA	Butylated hydroxyanisole
BHT	Butylated hydroxytoluene
BUN	Blood urea nitrogen
BW	Body weight
CK	Creatine kinase
DEHAMER	Experimental Animal Implementation and Research Center
EOSs	Eosinophils
GHS	Globally Harmonized System of Classification and Labelling of Chemicals
HCT	Hematocrit
HGB	Hemoglobin
HPLC	High-performance liquid chromatography
HPLC-DAD	High-performance liquid chromatography with diode-array detection
IMGs	Immature granulocytes
LDH	Lactate dehydrogenase
LYM	Lymphocyte
MCH	Mean corpuscular hemoglobin
MCHC	Mean corpuscular hemoglobin concentration
MCV	Mean corpuscular volume
MONs	Monocytes
MPV	Mean platelet volume
MTT	3-(4,5-Dimethylthiazol-2-yl)-2,5-Diphenyltetrazolium Bromide
NEUs	Neutrophils
NIH3T3	Mouse embryonic fibroblast cells
OECD	Organization for Economic Co-operation and Development
PCT	Procalcitonin
PDW	Platelet distribution width
PLCC	Platelet larger cell count
PLCR	Platelet larger cell ratio
PLTs	Platelets
RBCs	Red blood cells
RDV-CV	Coefficient of variation in red cell distribution width
SD	Standard deviation
TL	*T. longicaulis* subsp. *chaubardii*
UA	Uric acid
WBCs	White blood cells
